# *TBX21*, the Master regulator of the type 1 immune response, overexpresses in the leukocytes of peripheral blood in patients with late-onset Alzheimer’s disease

**DOI:** 10.1186/s12979-023-00385-1

**Published:** 2023-11-10

**Authors:** S. R. Fatemi Langroudi, M. Zeinaly, F. Ajamian

**Affiliations:** Department of Biology, Faculty of Sciences, University of Guilan, C.P., Namjoo St., Rasht, 41335-1914 Iran

**Keywords:** TBX21 (T-bet), Peripheral immunity, Late-Onset Alzheimer’s Disease (LOAD), Major Depressive Disorder (MDD), Expression

## Abstract

**Background:**

The involvement of the peripheral immune system in the etiology of neurodegenerative diseases has recently been emphasized. Genome-wide association studies (GWAS) have recently identified several candidate immune genes linked to development of both Alzheimer’s disease (AD) and depression. *TBX21 (T-bet)* which drives the Th1 immune response, is linked to the major depressive disorder (MDD) phenotype. This study investigated the association between the *TBX21* immune gene and the possibility of late-onset Alzheimer’s disease (LOAD) incidence in 194 LOAD and 200 control subjects using the real-time qPCR and the Tetra-ARMS-PCR methods. We also used an in silico approach to analyze the potential effects imparted by *TBX21 rs17244587* and *rs41515744* polymorphisms in LOAD pathogenesis.

**Results:**

We found that the *TBX21* “immune gene” had significantly elevated mRNA expression levels in the leukocytes of peripheral blood in patients with LOAD (*P* < 0.0001). We also found an upward trend in TBX21 expression with increasing age in LOAD patients compared to the control group (*P* < 0.05; CI = 95%). We noticed that the TT genotype of *rs41515744* plays a protective role in LOAD incidence, as it attenuates the expression of *TBX21* in the control group. We observed that the dominant model of *rs41515744* represented a substantial association with LOAD (*P* = 0.019).

**Conclusions:**

Our results show for the first time the likely impact of the *TBX21 (T-bet)* immune gene in LOAD development and that the elevated *TBX21* mRNAs in the WBCs of LOAD patients may represent a new easy diagnostic test for Alzheimer’s disease.

## Background

Alzheimer’s disease (AD), the most common form of age-related dementia, is a type of progressive neurodegenerative disorder and one of the leading causes of mortality-related dementia worldwide [1]. The hallmarks of AD are pathological neural loss and depletion, which arise from the formation of de novo amyloid-β (Aβ) plaques and *tau* neurofibrillary tangles (NFTs) in synaptic and cytoplasmic regions of neurons, respectively [2]. The main manifestattion of AD is cognitive failure leading to loss of memory [2].

Late onset Alzheimer’s disease (LOAD) often occurs in the late age of 60 s [3]. AD is a polygenic neurodegenerative disorder whose whole hereditary risk factors including related triggering signaling pathways remain to be discovered. Of the genes involved in AD pathology, the *ApoE* gene, is a crucial genetic cause of AD [4]. Other gene mutations that highly affect AD incidence are *APP*, *PSEN1* & *2*, *SORL1,* and *GAB2* [5].

The vital role of the innate immune system in developing AD is known. It initiates an inflammatory response in situ that ultimately activates microglia inside the brain, leading to the release of inflammatory cytokines such as IL-1β, IL-6, and TNF-α. The inflammatory response, therefore, seems to be a critical and powerful initiator of plaque formation in the brain, leading to neurodegeneration in LOAD [6]. In addition to the innate immune system, the involvement of peripheral immune inflammatory factors in the etiology of Alzheimer’s disease or other neurodegenerative diseases, such as major depressive disorder (MDD), has recently been addressed [7]. The inflammatory hypothesis of depression has widely been accepted by neuroscientists. The increased inflammatory activation of the immune system has been shown to play a vital role in the development of depression [8–10] and, the cytokine-induced neuroinflammation in major depressive disorder (MDD) has been well studied [11]. The fact that treatment of depression by antidepressants decreases inflammation in the brain strongly supports the immunopsychiatric link of depression [12]. As mentioned [7], both central (brain) and peripheral (innate) immune systems may play roles in such inflammation. A range of research already indicates that the inflammatory cytokines of macrophages in the body could reach the brain and this echo of inflammation in the brain can be linked to depression [8] and Alzheimer’s [13]. Genome-wide association studies (GWAS) have identified several immune candidate genes linked to the development of neurodegenerative diseases [14, 15]. Of those, the T-Box Transcription Factor 21 (*TBX21)* gene of the immune system functions in T lymphocytes and plays a role in NK cell maturation and functions [16]. *TBX21* (also called T-bet [T-box expressed in T cells]) is located on the 17q21.32 locus in humans and belongs to the T-box transcription factor (TF) family, which in T-helper-1 lymphocytes (Th1) encodes a T-bet transcription factor that establishes Th1 differentiation and maturation [16]. T-bet can then direct T-cell homing to proinflammatory sites and stimulate Th1 cells to secrete an interferon II called INF-G that causes inflammation in the site of release [17]. The T-bet transcription factor can also inhibit the polarization of some CD4^+^ T-cell subsets, such as Th2 or Th17 cells [18, 19]. Various single-nucleotide polymorphisms (SNPs) of *TBX21* affect the gene expression level of T-bet. For instance, *rs4794067* of *TBX21* is related to a higher risk of severity of asthma, risk of lupus erythematosus, and type 1 autoimmune hepatitis [20]. *TBX21* SNPs may correlate with some neurodegenerative diseases, such as systemic sclerosis [18]. The *TBX21* rs41515744, rs17244587 and rs2325717 polymorphisms have been linked to the major depressive disorder (MDD) phenotype [21]. According to this study, polymorphisms in many inflammatory-related genes including *TBX21*, *NR3C1* are associated with the risk of major depression and antidepressant response. The function(s) of *TBX21* or its various polymorphisms in neurodegenerative dementia, such as in AD, has not yet been studied. In this study, we not only studied two polymorphisms of the *TBX21* gene in LOAD incidence but also measured its expression alteration in white blood cells of LOAD patients and, investigated whether the changes in *TBX21* expression could link to the different genotypes of two *TBX21* polymorphisms.

We have previously shown elevated mRNA expression levels of the peripheral CD33 immune gene in WBCs of patients with LOAD [22]. Here, we investigated whether the expression of the *TBX21* immune gene is altered in the leukocytes of peripheral blood in LOAD patients, as it was for CD33 cases, to speculate whether it represents a LOAD-diagnosing biomarker. We then performed an in silico study to search the possible transcription factor (TF) binding sites on corresponding DNA motifs to determine which potential TF can possibly interact with the *TBX21* gene to derive its expression in LOAD patient Leukocytes. We also studied the two SNPs of *rs41515744* [C > T] and *rs17244587* [G > A] of the *TBX21* gene to find any potential correlation with them and LOAD incidence.

## Results

### *rs41515744* and *rs17244587* genotype frequency

Tetra-ARMS PCR results for *rs41515744* in healthy control samples showed 177 cases with CC, 18 cases with CT, and 5 cases with TT genotypes. The mined data from LOAD samples implied 155 cases with CC, 33 cases with CT, and 6 cases with TT genotypes. The genotype distribution for the *rs17244587* SNP for the healthy control individuals was 178 cases with GG, 12 cases with GA, and 10 cases with AA, while in LOAD patients, genotypes were 178 cases with GG, 6 cases with GA and 10 cases with AA. To study the frequency of various genotypes between the groups, a chi-square test was conducted. The results were as follows: *F* = 0.01; *P* = 0.96 for *rs41515744* which indicates that the difference is not significant (Table 1).

**Table 1:** Distribution of the genotype frequencies of *rs41515744* and *rs17244587* polymorphisms of the *TBX21* gene in LOAD patients (*n* = 194) and healthy control individuals (*n* = 200)

**SNP**	**Genotypes**	**LOAD patients, (*n* = 194), n (%)**	**Control, (*n* = 200), n (%)**	***F***	***P*-value**
**rs41515744**	CC	155 (78)	177 (88.5)	0.001	0.96
	CT	33 (17)	18 (9)		
	TT	6 (5)	5 (2.5)		
**rs17244587**	GG	178 (91.8)	178 (89)	0.29	0.59
	GA	6 (3.1)	12 (6)		
	AA	10 (5.1)	10 (5)		

In the genotype data of the *rs17244587* SNP, the statistical examination did not show significant correlations between the two patient and control groups (*F* = 0.29; *P* = 0.59) (Table 1).

In the next step, odd ratios (ORs) were analyzed to understand the effectiveness of *rs41515744* and *rs17244587* genotypes as risk factors in LOAD development. Based on the results of the OR test, the dominant model of *rs41515744* represented a significant association with LOAD with a *P*-value of 0.019 and an OR of 1.94 [1.11–3.39]. The codominant model of inheritance was at the margin of a significant level (*P* = 0.044), but this cannot be a reliable significant association (Table 2).

**Table 2:** The association between the *TBX21 rs41515744* polymorphism and LOAD (late-onset Alzheimer’s disease). *P*-values and ORs for the association are shown in dominant, codominant, and recessive models

**Group**	**Genotype (%)**			**Allele (%)**		**Dominant model**		**Co-dominant model**		**Recessives model**	
	CC	CT	TT	C	T	OR (95%CI)	*P*	OR (95%CI)	*P*	OR (95%CI)	***P***
**Case**	155 (79.8)	33 (17)	6 (3.1)	343 (88.4)	45 (11.6)	1.94 (1.11–3.39)	0.019	1.58 (1–2.48)	0.044	1.24 (0.37–4.15)	**0.72**
**Control**	177 (88.5)	18 (9)	5 (2.5)	372 (90.5)	28 (9.5)						

The number of subjects with each genotype and number of alleles (frequency in %)

ORs for different modes of inheritance were calculated using the Web-Assotest program

*OR* Odds ratio, *CI* Confidence interval

For the *rs17244587* polymorphism, the results showed no significant association between any models of inheritance and the risk of LOAD (P.0.05) (Table 3).

**Table 3:** Summary of *P*-values and ORs for the association between *TBX21 rs17244587* and LOAD in dominant, codominant, and recessive models

**Group**	**Genotype (%)**			**Allele (%)**		**Dominant model**		**Co-dominant model**		**Recessives model**	
	GG	GA	AA	G	A	OR (95%CI)	*P*	OR (95%CI)	*P*	OR (95%CI)	***P***
**Case**	178 (91.8)	6 (3.1)	10 (5.1)	362 (93.3)	26 (6.7)	0.73 (0.37–1.43)	0.35	0.89 (0.59–1.35)	0.59	1.03 (0.42–2.54)	**0.94**
**Control**	178 (89)	12 (6)	10 (5)	368 (92)	32 (8)						

The number of subjects with each genotype and number of alleles (frequency in %)

*OR* Odds ratio, *CI* Confidence interval, *LOAD* Late-onset Alzheimer’s disease

ORs for different modes of inheritance were calculated using the Web-Assotest program

### *rs41515744* and *rs17244587* allele frequency

Statistical analysis revealed that the frequencies of major (C) and minor (T) alleles of *rs41515744* in the LOAD group were 343 (84.4%) and 45 (11.6%), respectively. These allele frequencies in the control group were 372 (93%) (C) and 28 (7%) (T). The results for *rs17244587* indicated that the frequencies of major (G) and minor (A) alleles were 362 (93.3%) and 26 (6.7%) in the LOAD group and 368 (97%) and 32 (3%) in the control group, respectively.

Allele frequencies showed no significant association for either *rs41515744* (χ^2^ = 0.003 and *P* = 0.59) or *rs17244587* (χ^2^ = 0.005 and *P* = 0.56) polymorphisms (Table 4).

**Table 4:** Comparison of allelic frequencies between controls and LOAD patients for *rs41515744* and *rs17244587* SNPs in the *TBX21* gene

**SNP**	**Allele**	**Controls** **N (%)**	**Cases (LOAD)** **N (%)**	**χ**^**2**^	***P*-value**
*rs41515744*	C	372 (93)	343 (88.4)	0.003	0.59
	T	28 (7)	45 (11.6)		
*rs17244587*	G	368 (97)	362 (93.3)	0.005	0.56

### Increased *TBX21* expression in leukocytes in the peripheral blood of LOAD patients

**Fig. 1:**
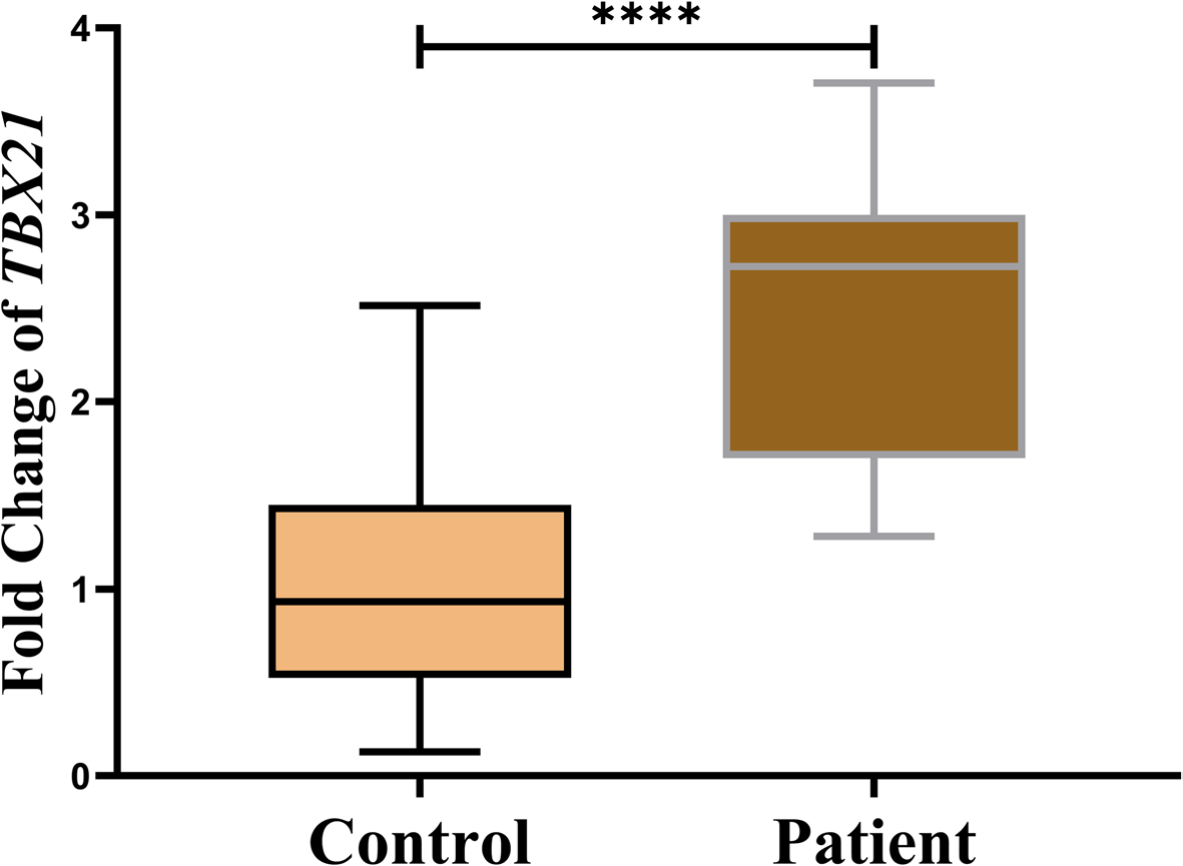
Evaluation of *TBX21* mRNA expression in the blood cells of LOAD patients. Relative *TBX21* mRNA expression is increased in WBCs from LOAD patients compared to those from control individuals. The total number of participants in the patient and control groups was 394**.** Data are normalized against GAPDH internal control. Bars represent mean values ± SDs. We used an unpaired *t test* to compare the different groups (****P < 0.0001)

Unpaired *t test* evaluations showed a significant increase (*P* < 0.0001) in *TBX21* expression levels in the leukocytes of peripheral blood of LOAD patients (mean values ± SDs: 2.44 ± 0.720) compared to normal healthy individuals (mean values ± SDs: 0.999 ± 0.633) (Fig. 1).

**Fig. 2:**
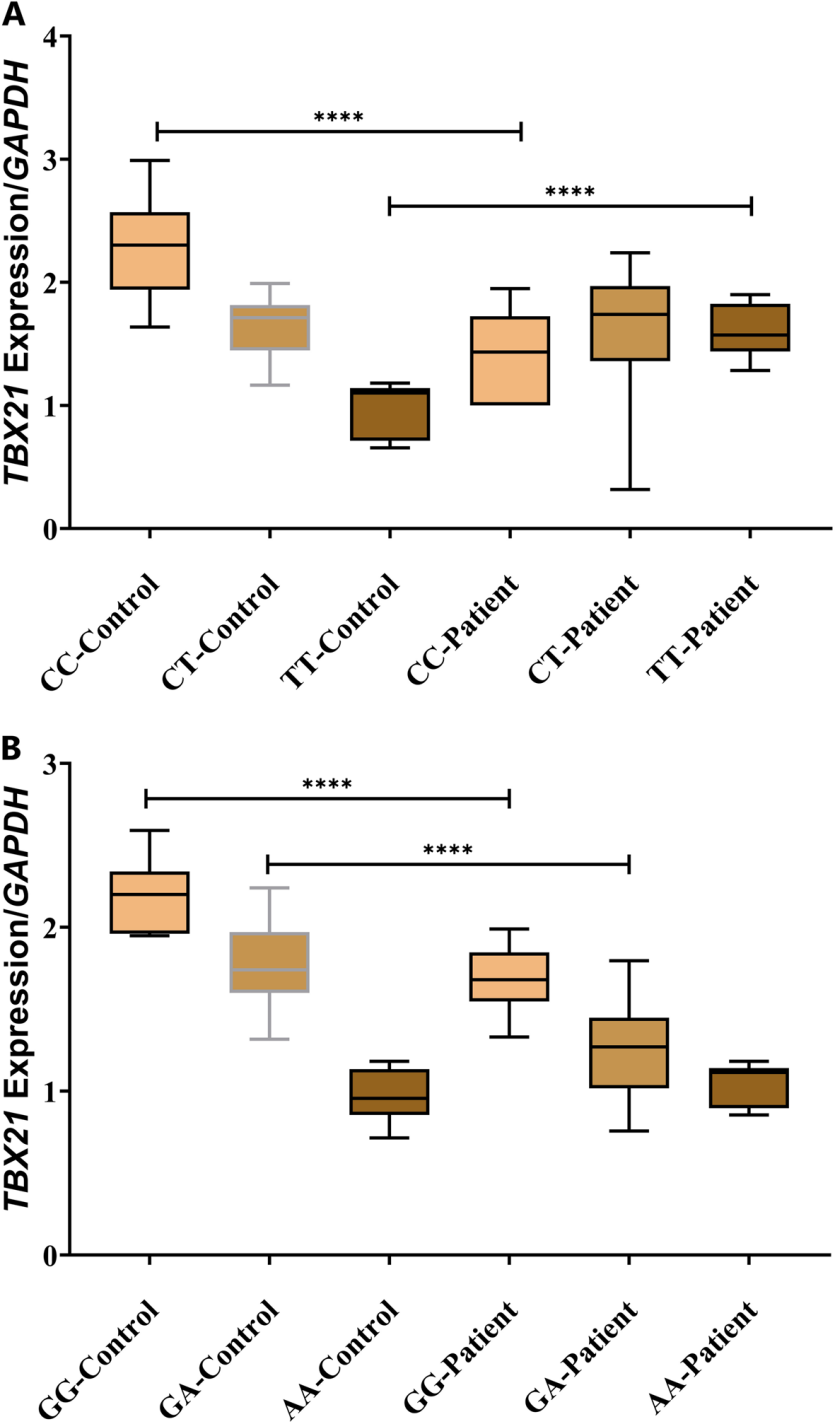
Relative levels of *TBX21* mRNAs in peripheral blood leukocyte samples of designated genotypes in **a** *rs41515744* polymorphism for genotypes of CC (155), CT (33), and TT (6) and in **b** *rs17244587* polymorphism for genotypes of GG (178), GA (6), and AA (10). (*n* = 194 in patients and *n* = 200 in control groups). The differences between groups are marked in the figure. The increase in *TBX21* mRNA levels was higher in the C/T of the *rs41515744* genotype than in the two other genotypes in the LOAD patients. The differences between LOAD and control samples in *TBX21* expression of the CC and TT genotypes are shown to be significant (****P < 0.0001). The increase in *TBX21* mRNA levels was higher in the G/G of the *rs17244587* genotype than in the two other genotypes in the LOAD patients. The differences between LOAD and control samples in *TBX21* expression of the GG and GA genotypes are shown to be significant (****P < 0.0001). Barres in box plots represent mean values ± SDs. We used a *Kruskal_Wallis test* to compare the different groups (****P < 0.0001). LOAD, late-onset Alzheimer’s disease

Next, we performed the expression evaluations of different genotypes for either studied *TBX21* SNP. The expression evaluation for the *rs41515744* SNP in LOAD patients indicated that the CC, CT, and TT genotypes had expression levels equal to 1.41 ± 0.36, 1.63 ± 0.53, and 1.60 ± 0.22, respectively. This showed that the CT genotype had the highest level of *TBX21* expression compared to the two other genotypes. The expression of different genotypes of the *rs41515744* SNP in the control cohort however, showed a different pattern compared to the case subjects: the expression of the CC, CT, and TT genotypes was equal to 2.29 ± 0.37, 1.65 ± 0.22 and 0.95 ± 0.23, respectively. The Kruskal–Wallis test revealed a significant relationship (*P* < *0.0001*) between *TBX21* expression in cases vs. controls for both CC, and TT genotypes whereas according to the results of *Dunn’s multiple comparisons test*, there was no significant relationship between CT (*p* > 0.05) genotypes in the two compared groups (Fig. 2a).

For the second polymorphism; the *rs17244587* SNP, the gene expression levels of various *TBX21* genotypes of GG, GA, and AA in LOAD patients were 1.69 ± 0.19, 1.25 ± 0.29, and 1.06 ± 0.12, respectively. Notably, the GG genotype of this *rs* has the highest expression among other genotypes. The expression of different genotypes of rs*17244587* in control groups showed almost the same similar pattern compared to the case subjects. The GG, GA, and AA genotypes in controls had expression levels equal to 2.17 ± 0.20, 1.78 ± 0.23, and 0.97 ± 0.16, respectively. The Kruskal-Wallis test revealed a significant relationship (*P* = 0.0001) between *TBX21* expression in cases vs. controls for both GG and GA genotypes. Based on the results of *Dunn’s multiple comparisons test*, there was no significant relationship between the AA (*P* > 0.05) genotype in the two compared groups (Fig. 2b).

**Fig. 3:**
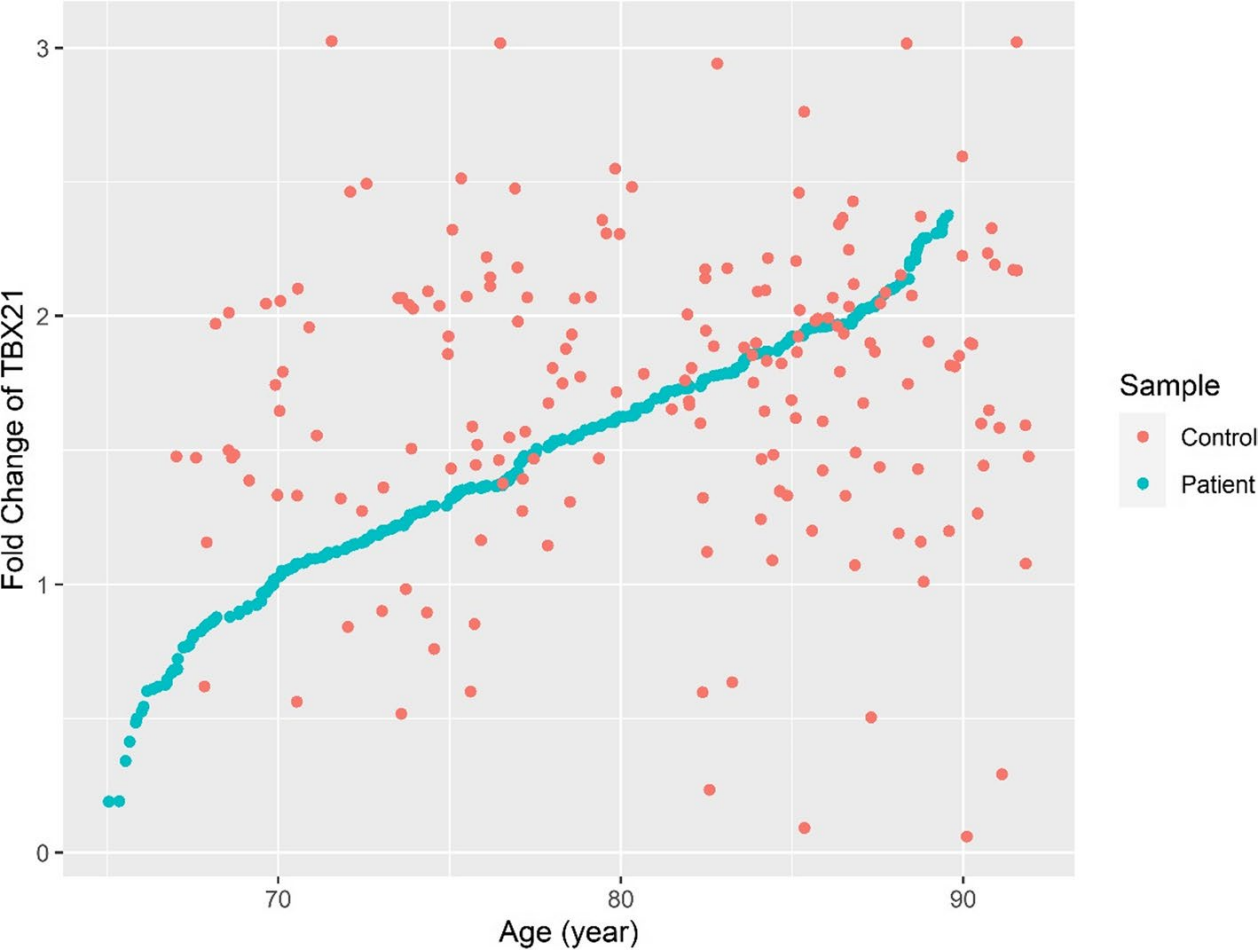
*TBX21* mRNA expression in peripheral blood leukocytes. Scatter plot of *TBX21* mRNA expression fold change against age in LOAD patients and control individuals. Each dot represents a sample. Blue dots and red dots are representatives of patients and controls, respectively

We also analyzed the expression levels of *TBX21* matching the increasing age of LOAD patients and control individuals. Interestingly, we found an upward trend in *TBX21* gene expression with increasing age in LOAD patients when compared to control individuals (*P* < 0.05; CI = 95%). A scatter plot of the fold change expression of *TBX21* versus the age of the studied population is shown in Fig. 3.

**Fig. 4:**
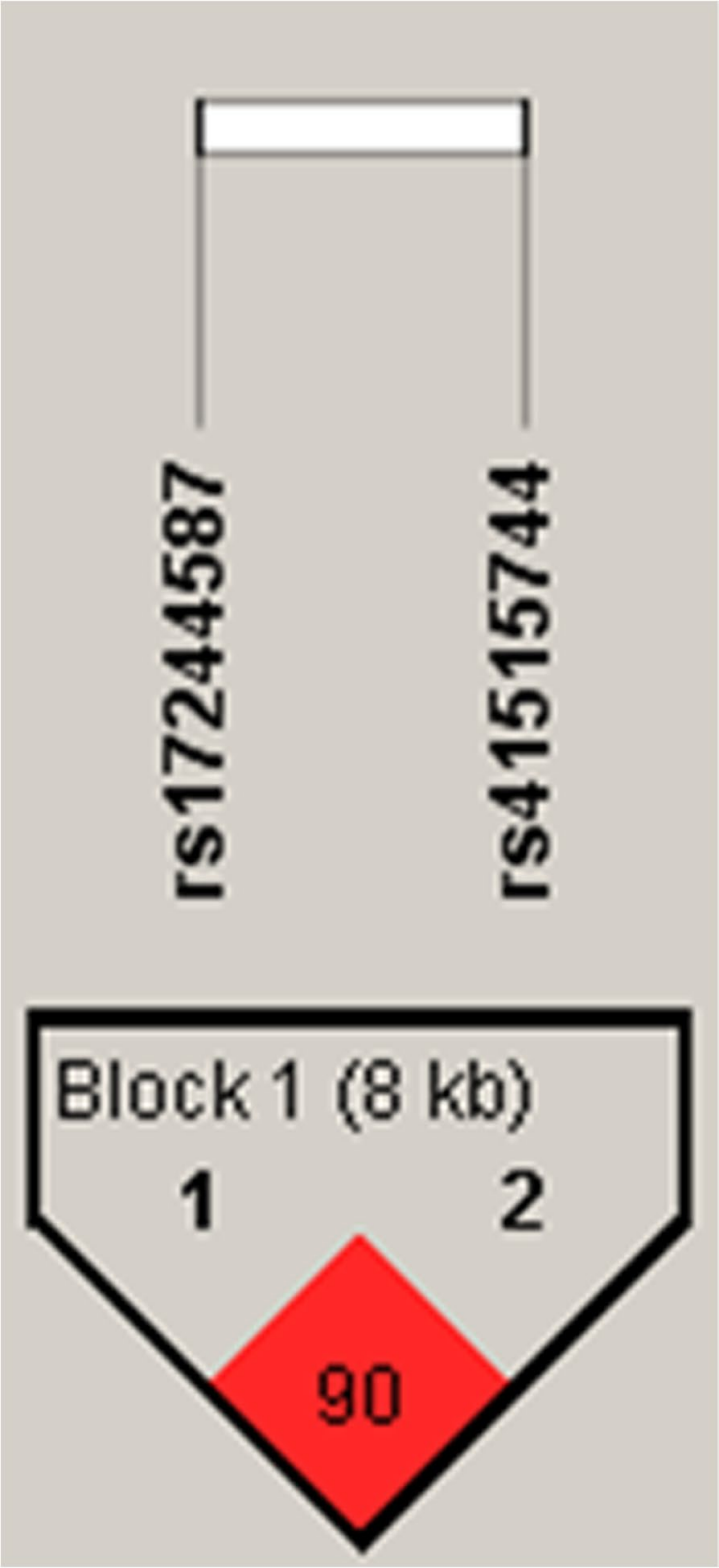
Schematic of haplotype block- formed by *rs17244587* and *rs41515744* SNPs in the *TBX21* locus

As shown in Fig. 4, there was a significant linkage disequilibrium (LD) between the pairwise haplotypes. The LD was 90 between *rs17244587* (G) and *rs41515744* (C), indicating a high linkage disequilibrium (*P* < 0.05) (Fig. 4).

The results showed that GC was the most frequent haplotype (0.93%), and the rarest haplotypes were GT (0.02%) in both case and control samples (Table 5). Based on the frequency of the pairwise haplotypes in cases and controls, none of the haplotypes conferred a risk or protective factor for LOAD susceptibility.

**Table 5:** Linkage disequilibrium analysis of *rs41515744* and *rs17244587 of the TBX21* locus

**Haplotype**	**Frequency (%)**	**Case, Control frequency**	***P*-value**	***P*-value (10,000 permutation)**
**GC**	0.929	0.913, 0.945	0.0301	0.0000E0
**AT**	0.044	0.042, 0.046	0.6925	0.1927
**GT**	0.023	0.045, 0.000	1.37e-7	0.1000

### In silico analysis for the *rs17244587* and *rs41515744* polymorphisms in the *TBX21* gene

We examined 15 bp upstream and 15 bp downstream of both SNPs using the *TFBIND software program* (http://www.tfbind.hgc.jp/) to investigate potential TF binding sites on corresponding DNA motifs. Generally, there are 3 models of binding for TFs in the identified ranges (see Table 6): 1) TFs that only bind to the dominant model (G for *rs17244587* and C for *rs41515744*); 2) TFs that only bind to the recessive model (A for *rs17244587* and T for *rs41515744*), and finally, 3) TFs that bind to either dominant or recessive models (G/A for *rs17244587* and C/T for *rs41515744*).

**Table 6:** In silico analysis of *TBX21 rs17244587* and *rs41515744* polymorphisms by the TFBIND program

**Polymorphism**	**Access Number**	**Factor**	**Allele**	**Strand**
***rs17244587* (G > A)**	M00086	V$IK1	G	+
	M00008	V$SP1	G	+
	M00098	V$PAX2	A	+
	M00192	V$GR	A	-
	M00212	V$POLY	A,G	+
	M00272	V$P53	A,G	-
	M00085	V$ZID	A,G	-
	M00146	V$HSF1	A,G	+
	M00147	V$HSF2	A,G	+
	M00255	V$GC	A,G	+
	M00175	V$AP4	A,G	-
	M00176	V$AP4	A,G	-
***rs41515744* (C > T)**	M00244	V$NGFIC	C	-
	M00108	V$NRF2	C	-
	M00184	V$MYOD	C	+
	M00033	V$P300	T	-
	M00200	V$CAAT	T	+
	M00131	V$HNF3B	T	+
	M00253	V$CAP	C,T	+
	M00255	V$GC	C,T	-
	M00105	V$CDPCR3	C,T	-
	M00196	V$SP1	C,T	-
	M00035	V$VMAF	C,T	-
	M00243	V$EGR1	C,T	-
	M00238	V$BARBIE	C,T	-
	M00127	V$GATA1	C,T	-
	M00011	V$EVI1	C,T	-

*V$* Refers to vertebrates

The bioinformatics analyses for the studied variants indicated that *rs41515744* was in the enhancer region and *rs17244587* in the enhancer flanking region of the *TBX21* gene (found on http://asia.ensembl.org/index.html). TFBIND results revealed that the transcription factors NGFIC, NRF2, and MYOD can bind only in the presence of the *rs41515744* major allele (C), while the minor allele T can create a new binding site for p300, CAAT, and HNF3B. Moreover, transcription factors, including IK1 and SP1, can only bind when the *rs17244587* major allele (G) is present and on the other hand, PAX2 and GR bind with the minor allele A.

## Discussion

Late-onset Alzheimer’s disease dementia is one of the most widespread age-related neurodegenerative disorders and is triggered by genetic, epigenetic, and environmental factors, and inflammation [1].

In this study, we show that the *TBX21* immunity gene has an overall > 2-fold elevated mRNA expression level in the leucocytes of peripheral blood in patients with late-onset Alzheimer’s disease (LOAD). *TBX21* has been shown to be involved in major depressive disorder (MDD) and the antidepressant response [21]. The increased expression observed to be associated with the presence of the TT genotype of the *rs41515744* SNP suggests that TT is a protective factor in healthy individuals as it attenuates TBX21 expression in control groups. We also found an upward trend in TBX21 expression in LOAD patients with increasing age compared to the control group (*P* < 0.05; CI = 95%). On the opposite side and, inside the LOAD groups, TBX21 could have fluctuated expression, depending on different genotypes. Our results conclusively indicated that the lower *TBX21* expression in LOAD patients is associated with the presence of GG and GA genotypes for *rs17244587* SNP and further, the CC genotype for *rs41515744* SNP. Our results also indicated that inside the studied population, there were no significant differences in either allelic or genotyping levels of *rs41515744* and *rs1244587*. Based on the OR test results, we found that the dominant model of the rs41515744 polymorphism represents a significant association with LOAD inheritance (0.019), while the codominant model of inheritance for this *rs* was only at the margin of a significant level (*P* = 0.044). In contrast, in the *rs17244587* SNP, there was no significant association between any model of inheritance and the risk of LOAD.

Recent findings indicate that a consistent in situ inflammation cascade has an enormous effect on the formation of Aβ plaques and NF tangles in both synaptic and cytoplasmic regions of neurons [2]. The triggering of “inside brain” inflammation processes, however, is not limited to the only activity of the brain’s own innate immune system. In fact, recent findings propose that such activity might also be the result of crosstalk between the internal environment of the brain and the peripheral immune system [7]. A genome-wide association study (GWAS) recently nominated several candidate “immunity genes” involved in brain inflammation and neurodegenerative disorders including Alzheimer’s disease [14], limbic-predominant age-related TDP-43 encephalopathy (LATE) [23], and major depressive disorder (MDD) [15]. We have previously shown that CD33, a transmembrane protein expressed on the surface of mature monocytes/macrophages [24], has strongly elevated mRNA expression levels in the leucocytes of peripheral blood in LOAD patients [22]. TBX21 of the T-box transcription factor family of T-helper-1 (Th1) lymphocytes encodes the T-bet transcription factor that directs T-cell homing to proinflammatory sites and stimulates Th1 cells to secrete INF-G to cause inflammation at the site of release [17]. The current study, to the best of our knowledge, is the first to study whether there is a possible connection between LOAD risk and changes in the expression of the *TBX21* inflammatory gene in peripheral leukocytes of LOAD patients. This study also investigates the possible difference in single nucleotide polymorphisms of *rs41515744* and *rs17244587* SNPs of the *TBX21* gene in LOAD cases compared to healthy individuals. Various studies have previously demonstrated the relationship between mutations in the *TBX21* gene and the development of different “immunity”-associated diseases, such as rheumatoid arthritis, asthma, type 1 diabetes called insulin dependent diabetes mellitus (IDDM), and systemic lupus erythematosus (SLE) [25]. In animal models of mice, studies have revealed that the TBX21 Th1-specific transcription factor affects the expression of various cytokines, especially INFγ [26]. Iinflammation has a crucial role in the formation of AD-related plaques and, INFγ is one of the most effective inflammatory factors whose chronic secretion stimulates inflammatory reactions by Th1 lymphocytes leading to tissue damage and degeneration as well as fibrosis [27]. Parnell GP and colleagues investigated the gene expression level of two distinct transcription factors encoded by *TBX21* and *EOMES* genes in T- and NK cells in patients with multiple sclerosis (MS). Their finding indicates that the expression level of both genes is decreased in MS patients’ T- and NK cells [26]. Our results, however, showed a > 2-fold increase in TBX21 transcript levels in WBCs from LOAD patients. The *TBX21* gene has remarkable polymorphisms based on the SNP database of NCBI (https://www.ncbi.nlm.nih.gov/snp/?term=TBX21). Gourh P et al. assessed independent data for the *rs11650354* SNP of the *TBX21* gene in 902 systemic sclerosis (SSc) patients compared to 4745 controls. Their results showed that the TT genotype of the *TBX21* gene had a recessive pattern for susceptibility to the SSc [28]. In addition, SSc patients with the CC genotype had higher expression levels of IL-2, IL-4, IL-5, and IL-13 (Th2), leading to altered cytokine balance and immune dysregulation compared to their control counterpart group. Gene expression profile analyses also revealed anomalies in interferon-I expression pathways in T cells for CC and TT genotypes of this SNP in SSc patients [28].

Depressive symptoms are common in Alzheimer’s patients and occur in approximately 20–30% of AD cases [29]. Depression is a serious disorder that affects approximately 300 million people worldwide. It could worsen existing medical conditions and increase functional disability [29, 30]. Clinical evidence suggests that depression might be related to AD status [31, 32], but it is not clear whether depression is a risk factor for AD, an early symptom of neurodegeneration, or a reaction to early cognitive impairment [32, 33]. Some findings suggest that depressive symptoms immediately follow the onset of AD rather than precede it [34]. Other authors suggest that the presence of depression in patients with AD raises the risk of behavioral disorders and accelerates functional decline [35]. Huden et al. reported that depression is the most consistent risk factor associated with behavioral or psychological symptoms and cognitive decline in Alzheimer’s patients [36]. Furthermore, several studies have concluded that depression at the end of life is associated with an increased risk of all causes of dementia, vascular dementia, and Alzheimer’s disease [37, 38], and late-life depression has been consistently associated with a twofold higher risk for dementia [39].

The team of Wong ML, in 2008, discovered the correlation between depression and two SNPs of *rs17244587* in *TBX21* and *rs2296840* in *PSMB4* genes in the American-Mexican population [21]. They found that having alleles with high-risk factors such as AA in *rs17244587* or TT in *rs2296840* will increase the risk of the development of major depressive disorder (MDD) [21]. As mentioned, LOAD patients often develop symptoms of depression. Very recently, Lutz MW et al. studied the genetic mechanisms underlying Alzheimer’s disease and major depressive disorder and suggested that MDD can be considered a prognosis of LOAD development [40]. Consistent with their results, we interestingly observed higher *TBX21* mRNA expression in leukocytes of LOAD patients than in control individuals. We also observed the higher *TBX21* mRNA expression was associated with the AA genotype of *rs17244587* in LOAD patients, although this difference was not statistically significant. Due to the differences in *TBX21* mRNA expression between patients and controls and to achieve a more precise view of the potential impacts influenced by *rs17244587* and *rs41515744* polymorphisms in *TBX21* expression in AD patients, we performed in silico subanalysis for additional investigation of the aforementioned polymorphisms. As will follow, our analysis indicates that a number of transcription factors (TFs) can bind to the site of *rs17244587* and, *rs41515744* polymorphisms to activate or inhibit transcription of *TBX21* in the presence of G, A, or G/A alleles for *rs17244587* and C, T, or C/T alleles for *rs41515744*. This may indicate that the *TBX21* “depressive” gene could play a role in the pathogenicity of AD from a molecular view:

Prior studies have demonstrated that SP1, p300, and NRF2 have an impact on AD [41–43]. Citron BA et al. previously found increased expression of the transcription factor Sp1 in Alzheimer’s patient brains, implying that transcription factors might be involved in disease pathogenesis [44]. In addition to its function in upregulating APP, Sp1 also controls COX-2, which can impact on how APP is processed and how amyloid is formed [45, 46]. Based on their results, patients with the GG genotype showed the highest rate of expression, which may be related to the binding site of the G allele compared to the lack of SP1 binding affinity in the presence of the A allele as the minor allele. It can be concluded that individuals with AA genotypes have a kind of protection against AD. NRF2 may prevent or delay the onset and progression of AD via different pathways, and drugs that activate NRF2 might be effective treatments for AD [42]. P300 may be responsive to the decline in language, memory, and executive abilities that have been seen in AD patients, according to Lee MS et al. [32]. Lee and his colleagues’ findings imply that P300 measures may be employed as physiologic indicators of diminished cognitive abilities in AD patients [43]. According to our current results, which showed that individuals with the CT genotype had the highest expression level, both NRF2 and p300 could bind to the region of *rs41515744* in a regulatory role. This can be interpreted as a dual role of the heterozygote genotype as the protector and inducer of AD. The CC genotype had the lowest rate of expression, indicating the protective role of FRF2.

## Conclusion

Collectively, our results show for the first time, the likely impact of the *TBX21 (T-bet)* immune gene in late-onset Alzheimer’s’ disease development and that the elevated *TBX21* transcripts in the leucocytes of peripheral blood in LOAD patients may represent a new easy diagnostic test for Alzheimer’s disease. An in silico study revealed that few transcription factors can potentially bind in the presence of either major or minor alleles of both *rs41515744* and *rs17244587* studied SNPs of the *TBX21 gene in LOAD cases. However,* we know this conclusion is not a direct finding of this study. This study was powered at 80% with a significance level of 5% confidence and a more extensive population analysis may give a more conclusive result. With the sample size used in this study, our result violates the assumption of Hardy-Weinberg equilibrium for the distribution of genotypes in both case and control groups, which indicates the limitation of this study. This violation may indicate a genotype misclassification of polymorphism results however, this would not affect the innovative finding of this study i.e. the overall overexpression of the TBX21 immune gene in the leucocytes of peripheral blood in LOAD patients compared to healthy individuals.

## Methods

### Participant recruitment and sample preparation

Eligibility criteria for the study population were set to the individuals older than 65, which subsequently were divided into two groups of patients with LOAD and healthy individuals matched for age and gender. Both LOAD and healthy individuals were diagnosed and identified by their physicians. A neurologist further double-examined each individual who preliminary was diagnosed with LOAD by a physician. The diagnosis of LOAD was performed based on the National Institute of Neurological Disorders and Stroke-Alzheimer’s Disease and Related Disorders Association (NINCDS-ADRDA) criteria (doi:10.1212/wnl.34.7.939). A total of 194 patients with LOAD (female:male, 136:58) and 200 control individuals (female:male, 139:51) were enrolled in this case-control study. The patients’ mean age ± standard deviation (SD) was 77.94 ± 8.642, ranging from 65 to 88 years. Controls with a mean age of 82.73 ± 8.395 years (ranging from 65 to 95 years) were non-AD diagnosed and healthy individuals. Patients and control individuals diagnosed with non-AD cognitive impairment, Parkinson’s disease, cardiovascular diseases, and diabetes were excluded from this study. All participants were of Persian ethnicity from or residing in northern Iran provinces; mostly from Guilan province. The blood samples were taken during 2 years of collection between December 2020 and December 2022 from several Disable and Elderly Hospice centers, mostly located in Rasht, the Guilan province’s capital. This study was approved by the University of Medical Sciences of Guilan’s Ethics Committee with international ID NO: IR.GUMS.REC.1399.427. A 5 ml peripheral blood sample of each individual with LOAD and/or healthy people was collected from several Disable and Elderly Hospice centers or elsewhere in Guilan Province, Rasht, Iran. All participants or their representatives provided informed consent. The blood samples were transferred to anticoagulant-containing EDTA K3 Venoject™ tubes *(Sinaclon, Iran)* by venipuncture procedure for further use.

### DNA isolation, total RNA extraction, and cDNA synthesis

Genomic DNA from the whole white blood cells (WBCs) of peripheral blood of individuals was extracted using Triton^®^ X-100 reagent (*Merck, Germany*) by the salting out standard method. Purified genomic DNA was stored at −20 °C until use. The extracted DNAs were used as templates for the *TBX21* genotyping study.

On the same day of sampling, total RNA from peripheral blood leukocytes of individuals was extracted using *TRIzol™* Reagent (*Invitrogen, USA*). The concentration and purity of extracted RNAs were evaluated by a NanoDrop device (*Thermo Scientific, USA*) at A260 and A280 nm wavelength ratios. The quality and integrity of RNAs were tested and approved by running RNA samples on gel electrophoresis. All RNAs were treated with DNase I (*Thermo Fisher Scientific, USA*) to ensure that there was no trace of potential genomic DNA contamination. cDNA synthesis (RT_PCR) was carried out by a cDNA synthesis kit (*Thermo Scientific, USA*) using 2 µg of total RNA, according to the manufacturer’s protocol. The rest of all the DNAs/synthesized cDNA and RNA were stored at -20°C and -80°C, respectively, for further use.

### Tetra-ARMS PCR and genotyping of SNPs

*TBX21* SNP genotyping was performed by multiplex amplification refractory mutation system polymerase chain reaction (Tetra-ARMS PCR). Two sets of inner and outer primers were precisely designed using Oligo7 software (*v7. 56*) presented in Table 7. Thereupon, all designed primers were tested on the platform of the NCBI primer BLAST tool to ensure that primers are specific and that no unintended PCR products will be amplified. All pairs of primers were synthesized by Metabion (*Metabion International AG, Germany*).

**Table 7:** Designed primer sequences for *rs41515744* [C > T] and *rs17244587* [G > A]

Primer (5’>3’)					Product size for:		
**rs41515744 [C>T]**			**GC (%)**	**Tm (**^**°**^**C)**	**C allele**	**T allele**	**Full product**
**Outer**	Forward	**5’- AATTGTCAGTACCATGCCTCAATCCCAG -3’**	46	60.5	185 bp	297 bp	424 bp
	Reverse	**5’- TGTAATCCCAACTACTCAGGAGGCTG -3’**	49	59.7			
**Inner**	Forward _(T allele)_	**5’- CATGTTGCTTAATCATGCACTACTCCGCT -3’**	44	60.7			
	Reverse _(C allele)_	**5’- AACAAGTTTTTGGAAACTAGAAAGCAGCCG -3’**	41	60			
**rs17244587 [G>A]**			**GC (%)**	**Tm (**^**°**^**C)**	**G allele**	**A allele**	**Full product**
**Outer**	Forward	**5’- TTCTCCTTTTGATAAGGAAGCTGAAGGAC -3’**	44	58.4	281 bp	384 bp	611 bp
	Reverse	**5’- TTTTCTCTGATCCCCACTGTGTTTGAGC -3’**	46.4	60			
**Inner**	Forward _(G allele)_	**5’- GGAGAAAAGAAGACAAGAAAGTCTTTGG -3’**	41	58.1			
	Reverse _(A allele)_	**5’- CACTAGATGCAAAAAGCTCCTTCAGGT -3’**	44.4	59			

The specific primers for *rs41515744* were a combination of F_outer_ + R_inner_ amplifying the normal allele of C with a product length of 185 bp and F_outer_ + R_inner_ amplifying the mutation of the T allele with a final product length of 297 bp. Additionally, the outer forward and reverse primers amplified the full *TBX21* selected region with a product length of 424 bp. In addittion, the specific primers of *rs17244587* were a combination of R_Outer_ + F_inner_ as well as F_outer_ + R_inner_ which amplify the normal G allele of 281 bp and mutant A allele of 384 bp, respectively. The combination of forward and reverse primers copies a 611 bp region of the gene.

All amplification reactions were run with a final volume of 25 µL reaction mixtures composed of a Master mix (*Ampliqon, Denmark*) of 12 µL, 1 µL of each primer, 5 µL of DNA template, and DEPC-treated water (4 µL). Thermal Cycler (*Bio-Rad, MJ Mini*, *USA*) program was set to initial denaturation (95°C- 5 min) followed by 40 cycles of a 3-step amplification program which consisted of denaturation (95°C- 45 secs), annealing (59°C for *rs17244587* and 65°C for *rs41515744* primers- 50 secs) and extension (72°C- 45 s) and settled by a final extension (72°C- 5 min) to avoid the synthesis of moiety nonspecific strands. Subsequently, electrophoresis was carried out via 2% agarose gel containing SafeStainTM and a DNA ladder (*SinaColon, Iran*).

### Quantitative real-time PCR (qPCR) and gene expression

The real-time quantitative PCR (qPCR) method was conducted in triplicate sample repeats in a final volume of 20 µL mixture including 10 µL of SYBR green PCR Master mix (*Ampliqon, no ROX, Denmark*), 1 µL of each forward and revers primer (with a concentration of 5 pmol/µL each), 1 µL of template cDNA (with an adjusted concentration of samples on 1 µg) and 7 µL of DEPC-treated water. The cycling conditions for the thermocycler (*Roche, LightCycler 96, Germany*) program were set up to one cycle of 96 °C for 10 min followed by 45 cycles of 95 °C for 30 s, and 60 °C for 30 s. All primers were designed again using Oligo7 software (*v7. 56*) and tested in the NCBI primer BLAST tool. An internal GAPDH control was used to measure the relative expression of the *TBX21* gene in each sample (Table 8). All pairs of primers were synthesized by Metabion (*Metabion International AG, Germany*). The annealing temperatures for all primers were obtained and optimized via the gradient PCR method in prestudy pilot testing. Eventually, the relative fold change in expression of the target *TBX21* and internal control (GAPDH) genes was calculated using the 2^−ΔΔCt^ cycle threshold method.

**Table 8:** Designed primer sequences for *TBX21* and *GAPDH* genes

**Gene**	**Primer**	**Sequence (5’ > 3’)**	**Length (nt)**	**GC (%)**	**Tm (**^**°**^**C)**	**Product size (bp)**
**TBX21** **NM_013351.2**	Forward	**5- GTGACCCAGATGATTGTGCTCC -3**	22	54.55	61.26	114
	Revers	**5- GATATGCGTGTTGGAAGCGTTG -3**	22	50.00	60.79	
**GAPDH** **NM_002046.7**	Forward	**5-CATCACCATCTTCCAGGAGCG-3**	21	57.14	60.81	235
	Revers	**5-GGAGGCATTGCTGATGATCTTG-3**	22	50.00	59.71	

### Statistical analysis

To examine the association between the *TBX21* polymorphisms and LOAD development, obtained results from ARMS-PCR were statistically analyzed to achieve chi-square (χ2), odds ratio (OR), *P*-value, and 95% Confidence Interval (CI) using WEB-Assotest (available at: http://ekstroem.com:3838/webassotest/). Analyses were also performed for dominant, codominant, recessive, and overdominant genetic models. Differences in allele frequency levels between age and gender were assessed by an independent t test and, a one-way ANOVA test that was used to evaluate the association between gender and age at genotype frequency levels. Significance was adjusted to *P*-values less than 0.05. Correspondingly, GraphPad/Prism software (v8.0.2) was used to apply *student t tests* on the qPCR data as well as one-way *ANOVA* for evaluations of various genotypes in each SNP. Linkage disequilibrium (LD) analysis was performed using Haploview (v4.2). The haplotype block for LD analysis has consisted of *rs41515744* and *rs17244587*.

## Data Availability

All data generated or analyzed during this study are included in this article, however, any supplementary material/data files can additionally be obtained from the corresponding author, Farzam Ajamian, Ph.D. Any other further inquiries can be directed to the corresponding author.
